# Distribution of tetraether lipids in sulfide chimneys at the Deyin hydrothermal field, southern Mid-Atlantic Ridge: Implication to chimney growing stage

**DOI:** 10.1038/s41598-018-26166-1

**Published:** 2018-05-23

**Authors:** Huaiming Li, Xiaoxia Lü, Chunhui Tao, Tianwei Han, Pengju Hu, Guoyin Zhang, Zenghui Yu, Chunming Dong, Zongze Shao

**Affiliations:** 1 Key Laboratory of Submarine Geoscience, Second Institute of Oceanography, State Oceanic Administration, Hangzhou, 310012 China; 2 State Key Laboratory of Biogeology and Environmental Geology, China University of Geosciences (Wuhan), Wuhan, 430074 China; 3 College of Marine Geosciences, Ocean University of China, 266100 Qingdao, China; 4 Key Laboratory of Marine Genetic Resources, Third Institute of Oceanography, State Oceanic Administration, 361005 Xiamen, China

## Abstract

This study presents analysis of four chimney samples in terms of glycerol dialkyl glycerol tetraether lipids (GDGTs), representing different growing stages of sulfide chimneys at the Deyin hydrothermal field, the southern mid-Atlantic ridge. The modified Bligh-Dyer method was used for lipid extraction and purification. GDGTs were analyzed with an Agilent 1200 series liquid chromatograph and 6460A triple quadrupole mass spectrometer. Our results showed that the intact polar GDGTs were more abundant than the core GDGTs in the 4 samples. The intact polar isoprenoidal GDGT-0 was the dominant composition (>70% of isoprenoidal GDGTs), indicating input of thermophilic *Euryarchaeota*. Most branched GDGTs were likely originated from the *in situ* thermophilic bacteria. However, the intact polar GDGTs in the sample at the late growing stage was similar to that in normal marine sediments, suggesting that the archaea mainly came from the planktonic *Thaumarchaeota* input. Our results suggested that the ratio of H-GDGTs to iGDGTs could be considered as a proxy to differentiated growing stages of a chimney. This study shed light on how to assess hydrothermal venting and sulfide chimneys in deep marine environments with a biomarker method in terms of different groups of GDGTs.

## Introduction

Hydrothermal sulfide chimneys growth may lead to variations in mineralogy and precipitation^1^. Relative abundance of Cu and Zn in a chimney may indicate different growing stage: Zn-rich usually for the mature stage with relative low fluids temperature (<300 °C), and Cu-rich for the early stage with high fluids temperature (>~300 °C)^1,2^. Hydrothermal venting and sulfides chimney growing processes may lead to a reduced environment rich in compounds such as H_2_, CH_4_, H_2_S and metal ions. Correspondingly, the microbial communities in a hydrothermal venting field are found to be different from those harbors in normal marine environments^3,4^. Furthermore, different growing stages of a chimney result in different microbial communities. This provides a new insight to assess sulfide chimneys with a biomarker method.

**Figure 1:**
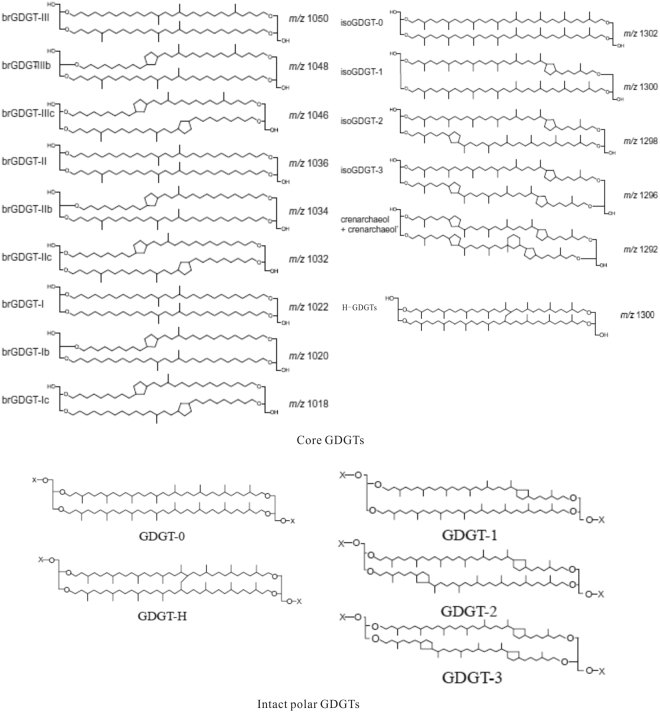
Structures of core and intact polar GDGTs.

Lipid biomarkers can provide crucial insights into the complex community structure of microorganisms and their metabolic status^5–8^. Glycerol dialkyl glycerol tetraether (GDGT) lipids (Fig. 1) are often used as biomarkers for archaea. In the last decades, the GDGTs in sedimentary environments have been used as a robust method to trace the paleo- marine and lacustrine environments and climate change^9–12^. Intact polar lipids (IPLs) refer to the lipids with polar head groups such as hexose and/or phosphate groups (Fig. 1)^13,14^, which are presumably transformed by cleavage of the head group into recalcitrant core lipids^15–17^. Usually, the intact IPLs are used to trace the living microbial organisms^14^. Recently, the glycol-IPLs were found more stable than what we presumed before, and the degradation kinetics of glycol-IPLs remains to be constrained, while the phospho-IPLs degraded rapidly with the death of the source organism^18,19^. Previous studies suggest that the phospho-IPLs are more applicable than the glyco-IPLs to trace the living source organisms^19^. The core GDGTs (C GDGTs) were detected in some active or inactive sulfate chimney samples^20,21^. Interestingly, the H-shaped GDGTs (H-GDGTs) were found to be rich in hydrothermal field and hot springs^21–23^. H-GDGTs have been proposed as indicators of thermophiles^20,21,24–26^ although they were also detected in some marine and lacustrine sediments^27,28^.

**Figure 2:**
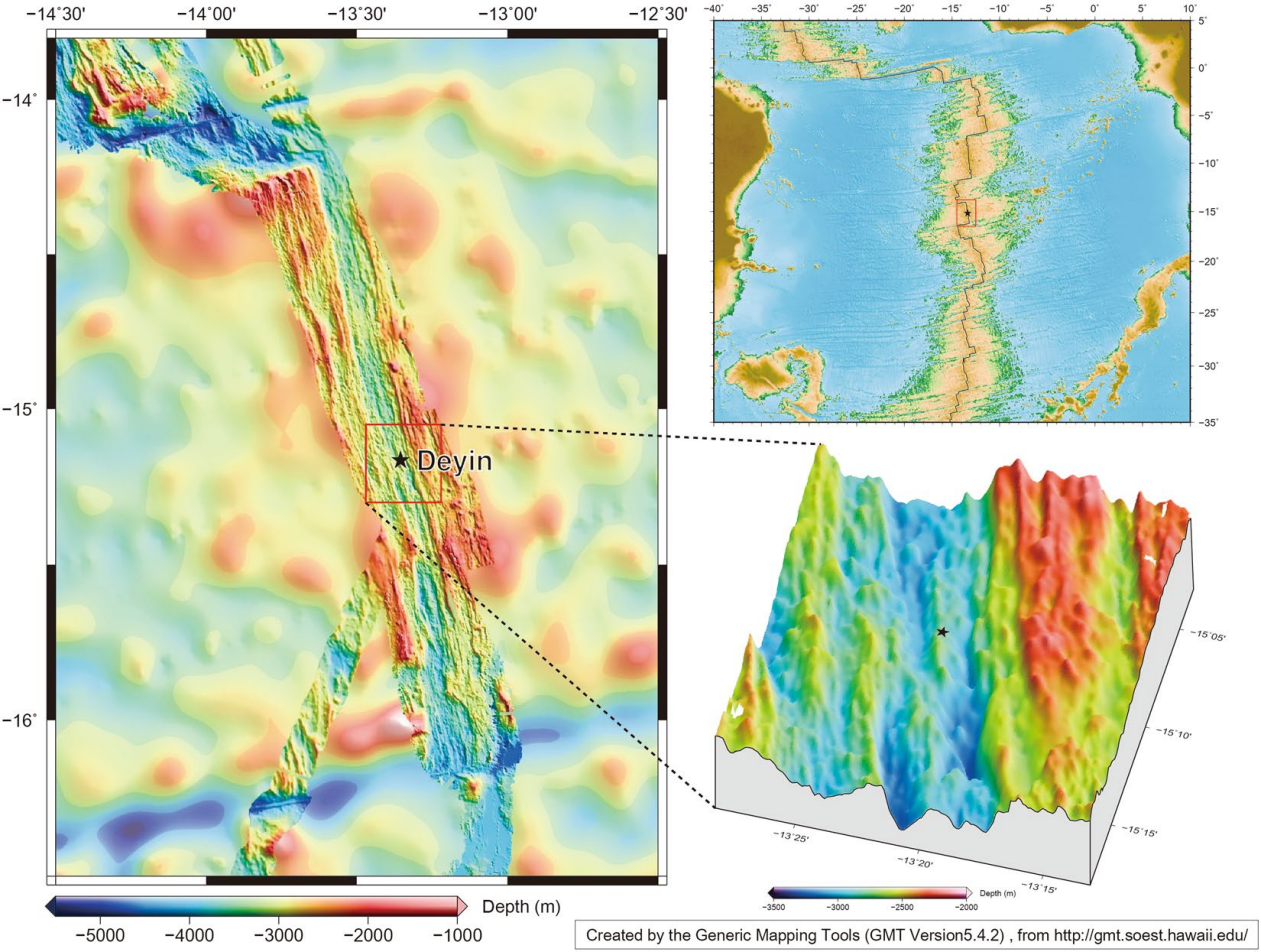
Sampling sites from SMAR (Created by the Generic Mapping Tools (GMT version 5.4.2), from http://gmt.soest.hawaii.edu/).

In this paper, we analyzed the lipids distribution in sulfide chimneys to distinguish the origins of lipids, and further discussed the lipid compositions at different growing stages of sulfide chimneys at the Deyin hydrothermal field (DHF). The DHF, with the depth of 2700 m, is located at the central ridge valley of the segment between Cardno and St. Helena Transform Faults, southern Mid-Atlantic Ridge (SMAR) (Fig. 2). Chimney samples were collected with a TV-Grab instrumented in the Chinese Dayang Cruise 26 (CDC26) for determining intact and core lipids. Those samples were immediately frozen at −80 °C after collection. The mineralogical characteristics of the samples were described in detail by Wang *et al*.^29^. In this study, four chimney samples (CS01, CS02, CS03, and CS04) were selected for analysis of intact and core lipids. The CS01 contains mainly pyrite and sphalerite suggesting low-temperature fluids mixing with the sea water at the early stage of a chimney growth. The CS02 is rich in pyrite and chalcopyrite, suggesting high-temperature with concentrated spray fluids mixing with sea water at the maturity stage of chimney growth. Another possibility is that the sample might be collected from the inner part of a chimney channel. The CS03 contains mainly pyrite, indicating the maturity stage of the chimney growth with a relatively low temperature. The CS04 has mainly amorphous oxides, usually shown in a long-term oxidizing environment in seawater at the extinct stage of a chimney.

## Results

### Distributions of core lipids

**Figure 3:**
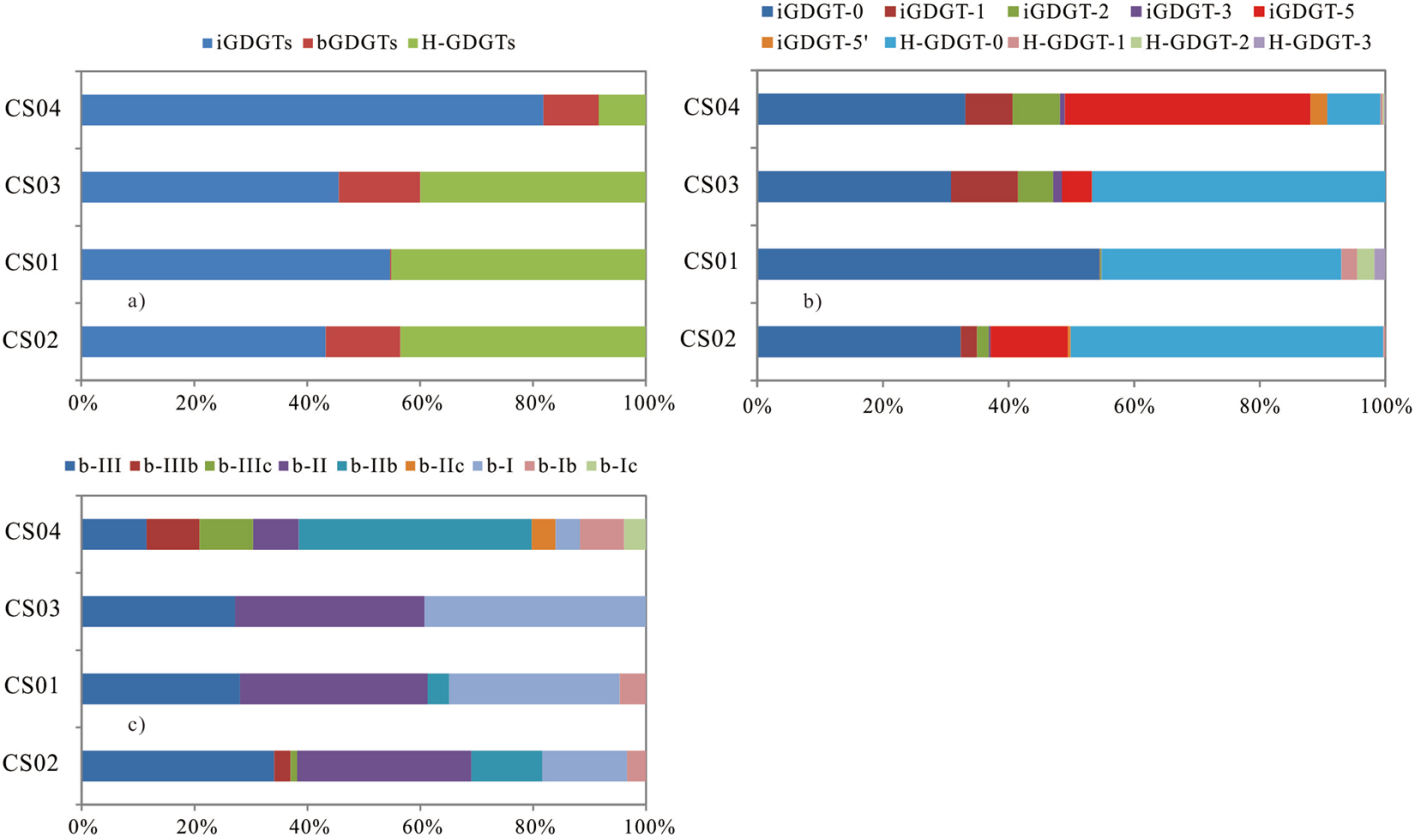
Fractional distribution of C GDGTs.

Almost all the C GDGTs could be detected in the four samples, but the concentrations varied. The concentration of C GDGTs was the highest in the CS04, followed by the samples from CS01, CS02 and CS03 (Supp. Table 1). Among all the detected C GDGTs, the iGDGTs and H-GDGTs were the predominant compositions except in the CS04, and the relative abundance of bGDGTs was the lowest (Fig. 3a). The fractional distribution of individual composition showed different in the four samples. The fractional distributions of normal iGDGTs and H-GDGTs were similar in the samples from CS02 and CS03, where the H-GDGT-0 was the predominant composition, followed by iGDGT-0 (Fig. 3b). In the CS01, the iGDGT-0 was the predominant composition followed by H-GDGTs. However, the crenarchaeol (iGDGT-5) and regio-isomer crenarchaeol could not be detected (Fig. 3b). The concentrations of H-GDGTs compositions were lower in the CS04 than in other samples. The distribution of iGDGTs was similar to those in a normal marine environment with relatively higher abundances of iGDGT-0 and iGDGT-5 (Fig. 3b). The predominant compositions of bGDGTs were bGDGT-I, bGDGT-II and bGDGT-III in the three samples, CS01, CS02 and CS03, while bGDGT-IIb was the predominant composition in the CS04 (Fig. 3c).

### Distribution of IPLs

**Figure 4:**
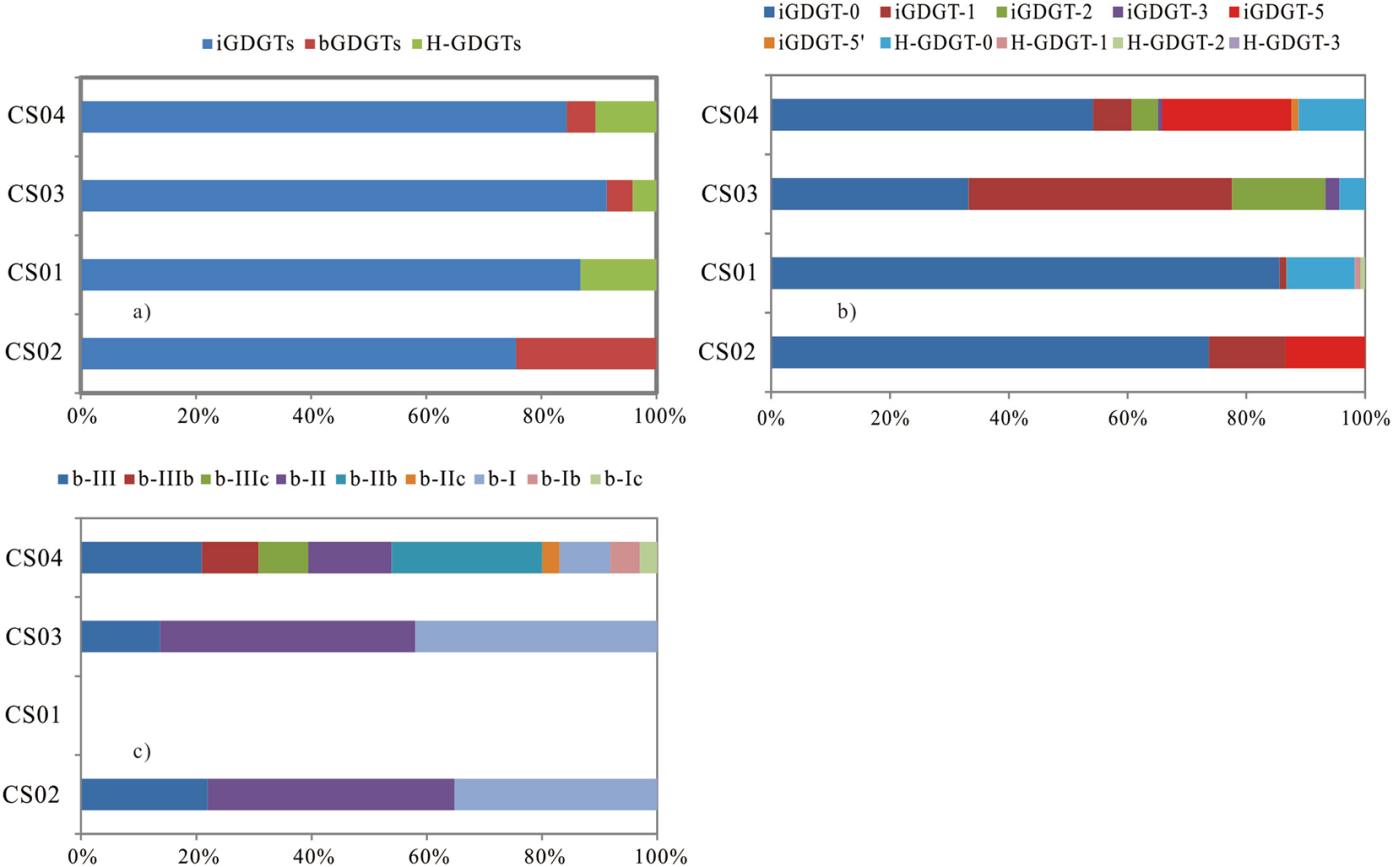
Fractional distribution of intact polar GDGTs.

Concentrations of the intact polar GDGTs (IPLs) were lower than those of C GDGTs. Concentration of the IPLs was the highest in the CS01, and followed by the samples from CS04, CS03 and CS02 (Supp. Table 1). The intact polar iGDGTs were the predominant compositions at all the samples. Intact polar bGDGTs were not detected from the CS01 while the intact polar H-GDGTs were identified from the CS02 (Fig. 4a). The intact polar iGDGT-0 was the predominant composition in the CS02, the CS03 and the CS04, while the intact polar iGDGT-1 and iGDGT-0 were the predominant compositions in the CS03. Interestingly, the intact polar crenarchaeol was detected in the CS01 and the CS03 (Fig. 4b). The components of the intact polar bGDGTs in the CS04 were mainly bGDGT-III, bGDGT-IIIb, bGDGT-IIIc, bGDGT-II and bGDGT-IIb, which were different from those in CS02 and the CS03 samples that bGDGTs were predominantly of bGDGT-I, bGDGT-II and bGDGT-III (Fig. 4c, Supp. Table 1).

## Discussion

### Origin of GDGTs

#### Isoprenoid GDGTs

The acyclic GDGT-0 is a common archaeal membrane lipid which may originate from methanogens^30,31^, mesophilic Group I *Crenarchaeota*^32^ and thermophilic *Crenarchaeota* and *Euryarchaeota*^33^. On the contrary, the crenarchaeol was thought to be mainly from ammonium-oxidizing *Thaumarchaeota*^32,34,35^. GDGT1-3 in most environments originates from *Crenarchaeota*, *Thaumarchaeota* and some *Euryarchaeota*^30,36–38^. In an environment with anaerobic oxidation of methane, especially where GDGTs 1-2 are dominant over the crenarchaeol, methanotrophic archaea of the ANME-1 phylogenetic cluster are considered as an important source of the GDGTs-1-3^30,39–41^. Recently, IPL distribution was found to be consistent with gene-based surveys, suggesting that the IPLs may be a good indictor to trace the microbial communities^5,8^.

The relative abundances of the intact polar GDGTs in the four samples appeared significantly different. The abundance of GDGT-1-3 is the highest in the CS03, up to 65.2% of intact polar GDGTs. The GDGT-1 was the predominant composition. In the CS01, GDGT-0 was the dominant composition, up to 98.6% of the detected intact polar GDGTs. In the CS02, GDGT-0 was the dominant composition, followed by crenarchaeol and GDGT-1. In the CS04, the intact polar iGDGT-0 was the dominant composition, followed by the intact polar crenarchaeol, GDGT-1, 2, regio-isomer crenarchaeol and GDGT-3. Interestingly, the regio-isomer was detected only in the CS04. Different distribution of the intact polar GDGTs in the four samples suggested the different microbial sources. The 16S rRNA analysis suggests that the archaea are the dominant microbial groups in the CS01 and the CS03 and the bacteria were the dominant group in the CS02 (Shao *et al*., unpublished data). In the CS03, *Euryarchaeota* (17%) and *Deltaproteobacteria* (14%) were the dominant groups. In the CS01, the most abundant groups were also affiliated to the phylum *Euryarchaeota* (54% of total sequence), including *Archaeoglobus* (23%), genus *Methanocaldococcus* (16%) and an unclassified genus (10%) within the order *Thermococcales*, as well as the phylum *Aquificae*, *Epsilonproteobacteria* and *Crenarchaeota*. In the CS02, the dominant groups were *Alphaproteobacteria* (12–23%) and *Nitrospirae* (12%) (Shao *et al*., unpublished data). Therefore, the intact polar GDGT-0-3 in the CS01 and the CS03 mainly originated from *Euryarchaeota*, especially in the CS01 where *Euryarchaeota* were the dominant microbial groups. The highest GDGT-1-3 in the CS03 might be due to the higher temperature in the dominant vent. On the contrary, bacteria were the predominant microbial groups in the CS02, and the composition of intact polar GDGTs was consisted with the results in an inactive silica-barite chimney from Loki’s Castle low-temperature venting field at the Arctic Mid-Ocean Ridge^42^ where the GDGTs were deduced from thermophilic *Crenarchaeota*^42^, which suggested that the GDGTs in the CS02 may be produced from thermophilic *Crenarchaeota* regardless of the hydrothermal venting temperature or the hydrothermal activity active or not. The special distribution of intact polar GDGT-1-3 might be a proxy to trace the dominant vent. The distribution of the intact polar GDGTs in the CS04 was similar to those in a normal oceanic environment, suggesting that the planktonic *Thaumarchaeota* may be the predominant composition of the microbial communities as well as the thermophilic *Euryarchaeota*.

#### H-shaped GDGTs (H-GDGTs)

H-shaped GDGTs were found to originate from the Euryarchaeota, including *Methanothermus fervidus*^24^, *Pyrococcushorikoshii*^25^, *Thermococcales*^26^, *Methanobactertherium mautotrophicus*^43^, and *Aciduliprofundum boonei*^27^, in hot springs and hydrothermal venting environments^26,44,45^. The H-GDGTs were generally considered to originate from thermophilic archaea^21,42^ though they could be detected in marine and lacustrine sediments with a low concentration (<6%)^27,28^. In the four samples, most intact polar H-GDGTs could not be detected due to the low organic carbon content. Concentrations of core H-GDGTs were as high as those of C GDGTs except in CS04 where H-GDGTs only occupied 10% of C-GDGTs (Supp. Table 1). Our results suggested that the H-GDGTs mainly were originated from the thermophilic archaea and the low proportion of H-GDGTs in CS04 might be due to dilution of the planktonic archaea.

#### Branched GDGTs

Branched GDGTs commonly existed in soils, peats, lacustrine sediments and marine sediments^46–50^ and were considered to originate from terrigenous *Acidobacteria*. However*, in situ* production in aquatic and sedimentary environments could not be excluded^51–54^. The bGDGTs were high in CS02, CS03 and CS04, and low in CS01 (Figs 3 and 4), consistent with the 16S rRNA result (Shao *et al*., unpublished data), which suggested the *in situ* hydrothermal bacteria contribution to bGDGTs. This finding is consistent with the results in Lost City hydrothermal field^21^.

### Microbial communities at the different growing stage of a sulfide chimney

The iGDGTs compositions were different in the four samples corresponding to different stages of a sulfide chimney growth. At the early stage, the fluid temperature was low due to the thoroughly mixing of hydrothermal fluids with seawater. At the maturity stage, the chimney was formed completely and the venting fluids could not mix well with the seawater thoroughly, which led to the fluid temperature high. The iGDGTs in CS01 sample at the early stage of the chimney growing were mainly composited by GDGT-0 and H-GDGTs with richer H-GDGTs (Fig. 3b, Table 1). CS02 and CS03 samples, at the maturity stage of the chimney growth where H-GDGTs were more abundant than iGDGTs, especially in CS02. Interestingly, the distribution of iGDGTs in CS04 was similar to that in normal marine sediment that GDGT-0 and GDGT-5 were the predominant composition of iGDGTs. This could be due to the fact that the archaea in water column deposited on the sulfide after the collapse of the chimney. Our result concluded that the iGDGTs composition could be as an indicator to the growing stage of a sulfide chimney.

**Table 1:** The relative abundance of H-GDGTs to total isoprenoidal GDGTs (in %).

Sample sites	CS02	CS01	CS03	CS04
H-GDGTs/(H-GDGTs + iGDGTs)	50.1	45.2	46.7	9.3

### Implications for GDGTs-based proxies

The TEX_86_ proxy was proposed to trace the sea surface temperature on the assumption that iGDGTs primarily originated from archaea lived in the water column^55^. Temperatures derived from the TEX_86_ at the four sites are 17.2 °C (CS02), 31.3 °C (CS01), 11.5 °C (CS03) and 31.2 °C (CS04) respectively. The mineralogical analysis showed that the sulfides were rich in Fe-Cu in the CS02, Fe-Zn in the CS01, and Fe in the CS03. Marcasite, a mineral in a low-temperature and high acidic condition^56^, was found only in CS01 sample. Temperature of the hydrothermal fluid in the CS01 was estimated to be lower than 350 °C. Temperatures of the hydrothermal fluid in the CS02 and the CS03 were estimated to be higher than 350 °C^56^. This suggests that the temperature obtained by TEX_86_ proxies represents archaea living temperature but not the temperature of the hydrothermal venting fluids.

**Figure 5:**
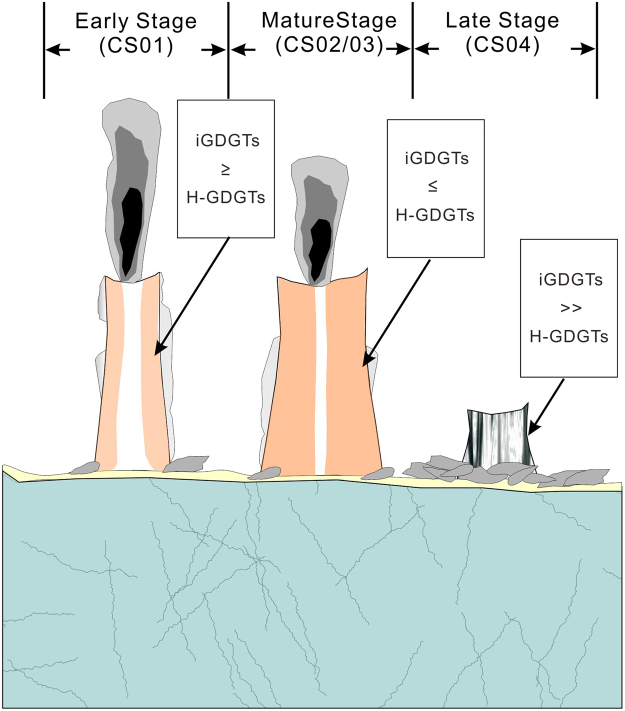
The correlation of lipids composition with chimney growing stage.

Occurrence of the H-GDGTs was proposed to be related to hydrothermal activity or hot springs. In hot springs from Yellowstone National Park, relative abundance of the H-GDGTs to the total iGDGTs was high in an acidic environment^57^ and was considered as another proxy for acidic environments. The relative abundance of H-GDGTs to total iGDGTs was high in the SMAR hydrothermal field except the hydrothermal oxide (CS04) (Table 1). This suggests that the abundance of H-GDGTs to total iGDGTs (>40%) might be as a proxy of hydrothermal activity. In addition, the relative abundance of H-GDGTs to iGDGTs in the four sites decreased according to the order of CS02, CS01, CS03 and CS04 where the temperature of hydrothermal fluids obtained from the mineral analysis decreased in sequence (Table 1)^29^, indicating that it could be used to trace the temperature of hydrothermal fluid at different growing stage of a chimney (Fig. 5). Interestingly, we found that the concentrations of GDGTs (including normal GDGTs and H-GDGTs) were higher in the CS01 and the CS04 than in the CS02 and the CS03 (Supp. Table 1), suggesting that archaea were more abundant in hydrothermal venting fields with a low temperature.

## Conclusions

Three groups of C GDGTs and intact polar GDGTs were identified in the four samples from the hydrothermal field in the southern Mid-Atlantic Ridge (SMAR). Our analysis showed that isoprenoid GDGTs (including H-GDGTs) were the predominant compositions in both the C GDGTs and the intact polar GDGTs. The intact polar iGDGT-0 was the dominant composition in the CS01, the CS02 and the CS03 due to the thermophilic Euryarchaeota input. Crenarchaeol was detected in the CS02 and CS03 because of the thermophilic Thaumarchaeota input. The difference in the GDGTs distribution pattern was likely due to the different microbial communities at the four sites. The distribution of intact polar GDGTs in the CS04 different from those in other three samples was similar to that in normal ocean sediments, indicating planktonic Thaumarchaeota input. Our results indicated that most bGDGTs originated from the *in situ* thermophilic bacteria. The relative abundance of H-shaped GDGTs to isoprenoidal GDGTs was high (>45%) in the hydrothermal field, which suggested the value of H-GDGTs/iGDGTs could be used to infer the temperature of hydrothermal fluid and also as an index to different growing stage of a chimney.

## Methods

### Lipid extraction and purification

Aliquots of samples were extracted using a modified Bligh-Dyer method^58,59^: firstly with a mixture of K_2_HPO_4_ (50 mmol/l, pH 7.4): MeOH: DCM = 4: 10:5 ultrasonically for 15 min 4 times, and then with the DCM ultrasonically 2 times. All the extracted liquids were combined into a separate funnel, rinsed with distilled ionic water after the DCM. The DCM phases containing the extracted lipids were collected into a round-bottomed flask and carefully evaporated to dry under a nitrogen stream below 40 °C. The total lipid extract (TLE) was further fractionated in a vial silica gel column using a slightly modified version of the separation procedure developed by Oba *et al*.^60^ and Tierney *et al*.^12^. TLEs were eluted to provide the portion containing the core GDGTs(C GDGTs) with hexane: EtOAc (3: 1) and the portion containing the IPL compounds with MeOH. The portion for analyzing the C GDGTs was further dried with N_2_ gas and stored at −20 °C until analysis. The portion for analyzing the IPLs compounds was subjected to the acid-catalyzed hydrolysis to cleave polar head groups by adding 20 mL of 5% HCl in MeOH and refluxing heating for 2.5 h. The solution was cooled to room temperature and adjusted the pH value to 5 with addition of 1 mol/l KOH in MeOH, and then added bi-distilled water to a volumetric ratio of H_2_O to MeOH at 1:1. The mixture was washed six times with the DCM, and then dried with N_2_ gas, and then stored at −20 °C for analysis.

### HPLC/MS analysis

Aliquot of the prepared samples were dissolved in 300 μl hexane: isopropanol (99:1), with C_46_ glycerol trialkyl glycerol tetraether (GTGT) added as internal standard. GDGTs were analyzed using an Agilent 1200 series liquid chromatograph and 6460A triple quadrupole mass spectrometer equipped with an autosampler and ChemStation manager software. An aliquot of sample (10–30 µl) was injected and separation was achieved with an Alltech Prevail Cyano column (150 mm × 2.1 mm, 3 µm; Grace, Deerfield, IL, USA). The elution gradient followed Schouten *et al*.^61^ with some modifications. GDGTs were eluted isocratically in the first 5 min with A/B 9:1, where A = hexane and B = hexane: isopropanol (9:1). The following linear gradient was then used: 90/10 A/B to 82/18 A/B from 5 to 45 min, followed by 100% B (10 min) to wash the column and then 90/10 A/B to equilibrate it. GDGTs were detected using selected ion monitoring (SIM), targeting *m/z* 1302, 1300, 1298, 1296, 1292, 1050, 1048, 1046, 1036, 1034, 1032, 1022, 1020, 1018, 653, and 744. Relative abundances were determined by peak area integration of [M+H^+^ in the extracted ion chromatogram. The relative abundance of an individual GDGT is defined as percentage of total iGDGTs or bGDGTs.

## Electronic supplementary material

Supplementary Table 1
