# Supplementary material for: Distribution of tetraether lipids in sulfide chimneys at the Deyin hydrothermal field, southern Mid-Atlantic Ridge: Implication to chimney growing stage
Source: Sci Rep. 2018 May 23;8:8060. doi: 10.1038/s41598-018-26166-1 (PMC5966438; doi:10.1038/s41598-018-26166-1)
Supplement: Supplementary file 1 — Supplementary Table 1 [file 41598_2018_26166_MOESM1_ESM.doc]

Distribution of tetraether lipids in sulfide chimneys at the Deyin hydrothermal field, southern Mid-Atlantic Ridge: Implication to chimney growing stage

Huaiming Li^1†^, Xiaoxia Lü^2†^*, Chunhui Tao^1^, Tianwei Han^2^, Pengju Hu^2^, Guoyin Zhang^1^, Zenghui Yu^3^, Chunming Dong^4^, Zongze Shao^4^

1. Second Institute of Oceanography, State Oceanic Administration (SOA), Hangzhou, 310012 China
2. State Key Laboratory of Biogeology and Environmental Geology, China University of Geosciences (Wuhan), Wuhan, 430074, China
3. College of Marine Geosciences, Ocean University of China, 266100, Qingdao, China
4. Key laboratory of marine genetic resources of SOA, Key laboratory of marine genetic resources of Third Institute of Oceanography, State Oceanic Administration (SOA), 361005, Xiamen, China

^†^ These authors contributed equally to this work.

* Corresponding author: [luxiaox@163.com](mailto:luxiaox@163.com) (X. Lü)

Sup. Table 1 The concentrations and relative abundances of GDGTs in the samples from SMAR hydrothermal fields

|  |  | sample | iGDGT-0 | iGDGT-1 | iGDGT-2 | iGDGT-3 | iGDGT-5 | iGDGT-5' | H-GDGT-0 | H-GDGT-1 | H-GDGT-2 | H-GDGT-3 | b-III | b-IIIb | b-IIIc | b-II | b-IIb | b-IIc | b-I | b-Ib | b-Ic | iGDGTs | bGDGTs | H-GDGTs |
| --- | --- | --- | --- | --- | --- | --- | --- | --- | --- | --- | --- | --- | --- | --- | --- | --- | --- | --- | --- | --- | --- | --- | --- | --- |
| Concentration  (ng/g) | C GDGTs | CS02 | 4.48 | 0.36 | 0.26 | 0.04 | 1.70 | 0.06 | 6.88 | 0.05 | n.d. | n.d. | 0.72 | 0.06 | 0.02 | 0.65 | 0.27 | n.d. | 0.32 | 0.07 | n.d. | 6.89 | 2.11 | 6.93 |
|  |  | CS01 | 381.35 | 0.61 | 1.44 | 0.36 | n.d. | n.d. | 267.25 | 17.76 | 19.10 | 12.25 | 0.36 | n.d. | n.d. | 0.43 | 0.05 | n.d. | 0.39 | 0.06 | n.d. | 383.77 | 1.28 | 316.37 |
|  |  | CS03 | 0.79 | 0.27 | 0.14 | 0.04 | 0.12 | n.d. | 1.19 | n.d. | n.d. | n.d. | 0.12 | n.d. | n.d. | 0.14 | n.d. | n.d. | 0.17 | n.d. | n.d. | 1.36 | 0.43 | 1.19 |
|  |  | CS04 | 1064.17 | 241.54 | 242.57 | 25.43 | 1255.82 | 85.78 | 271.60 | 11.60 | 8.59 | 5.45 | 40.10 | 32.53 | 33.09 | 28.19 | 143.56 | 14.66 | 15.02 | 27.22 | 13.50 | 2915.30 | 347.88 | 297.24 |
|  | IPLs | CS02 | 0.34 | 0.06 | n.d. | n.d. | 0.06 | n.d. | n.d. | n.d. | n.d. | n.d. | 0.03 | n.d. | n.d. | 0.06 | n.d. | n.d. | 0.05 | n.d. | n.d. | 0.47 | 0.15 | n.d. |
|  |  | CS01 | 220.57 | 2.90 | 0.16 | 0.03 | n.d. | n.d. | 29.64 | 2.37 | 2.00 | n.d. | n.d. | n.d. | n.d. | n.d. | n.d. | n.d. | n.d. | n.d. | n.d. | 223.66 | n.d. | 34.01 |
|  |  | CS03 | 1.28 | 1.70 | 0.60 | 0.09 | n.d. | n.d. | 0.17 | n.d. | n.d. | n.d. | 0.02 | n.d. | n.d. | 0.08 | n.d. | n.d. | 0.08 | n.d. | n.d. | 3.67 | 0.18 | 0.17 |
|  |  | CS04 | 31.17 | 3.73 | 2.55 | 0.40 | 12.55 | 0.68 | 6.44 | n.d. | n.d. | n.d. | 0.63 | 0.29 | 0.26 | 0.43 | 0.78 | 0.09 | 0.26 | 0.15 | 0.09 | 51.07 | 2.99 | 6.44 |
| Relative  abundance  (%) | C GDGTs | CS02 | 64.93 | 5.23 | 3.70 | 0.65 | 24.66 | 0.83 | 99.31 | 0.69 | - | - | 10.37 | 2.89 | 1.16 | 30.85 | 12.59 | - | 14.99 | 3.39 | - |  |  |  |
|  |  | CS01 | 99.37 | 0.16 | 0.38 | 0.09 | - | - | 84.48 | 5.61 | 6.04 | 3.87 | 0.11 | - | - | 33.21 | 3.84 | - | 30.21 | 4.65 | - |  |  |  |
|  |  | CS03 | 57.89 | 20.00 | 10.45 | 2.71 | 8.95 | - | 100.00 | - | - | - | 9.80 | - | - | 33.59 | - | - | 39.23 | - | - |  |  |  |
|  |  | CS04 | 36.50 | 8.29 | 8.32 | 0.87 | 43.08 | 2.94 | 91.37 | 3.90 | 2.89 | 1.83 | 13.49 | 9.35 | 9.51 | 8.10 | 41.27 | 4.21 | 4.32 | 7.83 | 3.88 |  |  |  |
|  | IPLs | CS02 | 73.71 | 12.89 | - | - | 13.40 | - | - | - | - | - | 21.99 | - | - | 42.84 | - | - | 35.18 | - | - |  |  |  |
|  |  | CS01 | 98.62 | 1.30 | 0.07 | 0.02 | - | - | 87.14 | 6.97 | 5.88 | - | - | - | - | - | - | - | - | - | - |  |  |  |
|  |  | CS03 | 34.77 | 46.33 | 16.45 | 2.45 | - | - | 100.00 | - | - | - | 14.85 | - | - | 44.30 | - | - | 41.97 | - | - |  |  |  |
|  |  | CS04 | 61.04 | 7.31 | 4.98 | 0.78 | 24.57 | 1.32 | 100.00 | - | - | - | 9.73 | 9.82 | 8.66 | 14.47 | 26.12 | 2.98 | 8.79 | 5.11 | 3.09 |  |  |  |
